# Localization System for Lightweight Unmanned Aerial Vehicles in Inspection Tasks

**DOI:** 10.3390/s21175937

**Published:** 2021-09-03

**Authors:** Diego Benjumea, Alfonso Alcántara, Agustin Ramos, Arturo Torres-Gonzalez, Pedro Sánchez-Cuevas, Jesus Capitan, Guillermo Heredia, Anibal Ollero

**Affiliations:** 1 GRVC Robotics Laboratory, University of Seville, 41004 Sevilla, Spain; dbgayango@us.es (D.B.); aamarin@us.es (A.A.); aramos11@us.es (A.R.); arturotorres@us.es (A.T.-G.); pedro.sanchezcuevas@uni.lu (P.S.-C.); jcapitan@us.es (J.C.); aollero@us.es (A.O.); 2 Space Robotics Research Group, SnT—University of Luxembourg, 1855 Luxembourg, Luxembourg

**Keywords:** Unmanned Aerial Vehicles, localization, infrastructure inspection

## Abstract

This paper presents a localization system for Unmanned Aerial Vehicles (UAVs) especially designed to be used in infrastructure inspection, where the UAVs have to fly in challenging conditions, such as relatively high altitude (e.g., 15 m), eventually with poor or absent GNSS (Global Navigation Satellite System) signal reception, or the need for a BVLOS (Beyond Visual Line of Sight) operation in some periods. In addition, these infrastructure inspection applications impose the following requirements for the localization system: defect traceability, accuracy, reliability, and fault tolerance. Our system proposes a lightweight solution combining multiple stereo cameras with a robotic total station to comply with these requirements, providing full-state estimation (i.e., position, orientation, and linear and angular velocities) in a fixed and time-persistent reference frame. Moreover, the system can align and fuse all sensor measurements in real-time at high frequency. We have integrated this localization system in our aerial platform, and we have tested its performance for inspection in a real-world viaduct scenario, where the UAV has to operate with poor or absent GNSS signal at high altitude.

## 1. Introduction

**Figure 1:**
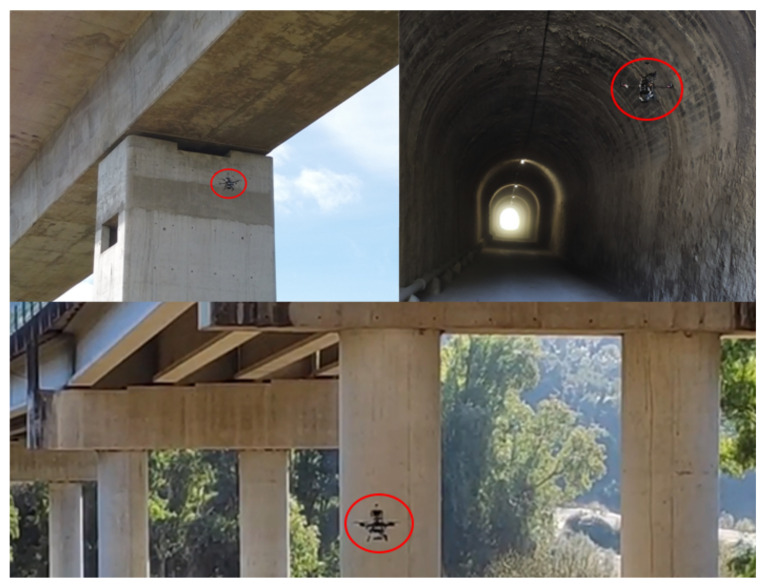
Example images of a UAV (circle) inspecting different civil infrastructures, such as a viaduct (**top left**), a tunnel (**top right**), and a bridge (**bottom**).

Over the last decade, the use of Unmanned Aerial Vehicles (UAVs) has been continuously growing for a broad spectrum of applications [1,2]. In some of these applications, UAVs collect data using cameras or sensors, e.g., for filming or remote sensing [3,4,5,6], whereas in others, they are equipped with end-effectors to accomplish aerial manipulation tasks [7,8,9,10]. More specifically, UAVs are of particular interest in inspection tasks of civil infrastructures [11]. Bridges, tunnels, or viaducts need to be periodically inspected to assess their health and durability (see Figure 1); UAVs are particularly suitable for this task due to their maneuverability and capacity to access complex locations [12,13,14]. Additionally, UAV-based inspection is cheaper and faster than classic inspection methods. Thus, UAVs could enable the continuous assessment of the infrastructure and the improvement of the preventive maintenance program, reducing long-term maintenance costs.

The operational conditions in those inspection environments can be quite challenging, as the operation is usually performed at relatively high altitude (tens of meters) in confined spaces or close to the infrastructure itself, the UAV could be Beyond Visual Light Of Sight (BVLOS), and the signal for the GNSS (Global Navigation Satellite System) localization systems may be unreliable, and so on. Therefore, in order to reduce risks and global costs, it is required to have a UAV localization system that is reliable and that works robustly in all situations, even when the coverage from GNSS satellites is poor. Moreover, a global localization that is *time-persistent* is necessary from an application point of view. This means that the UAV should be capable of revisiting a specific inspection point in two different flights, using exactly the same global coordinates in order to re-evaluate the defects that were previously identified. To sum up, the system needs to fulfill the following specific operational requirements:

- *Defect traceability*: The system should provide a state estimation of the UAV with respect to a fixed and time-persistent reference frame. This enables the capability of tracing the evolution of the detected defects properly throughout subsequent inspection operations.
- *Accuracy*: In the target inspection operations, accurate localization is essential. On the one hand, the system should be able to revisit any specific defect that was previously detected. On the other hand, precise localization is needed to operate the platform safely since this kind of operation usually requires the UAV to fly quite close to the infrastructure being inspected and even to make physical contact with it.
- *Reliability*: The UAV state estimation should consist of a constant stream at high frequency, coming from the fusion of multiple available sensors, in order to increase the localization robustness against possible inaccuracies.
- *Fault tolerance*: The system should react to malfunctioning components. If one of the sensors fails, the system should be capable of detecting this malfunction and taking actions to discard measurements from that sensor, ensuring the integrity of the final localization.

In this paper, we present a UAV localization system for large infrastructure inspection applications. The main objective is to design a system whose localization is reliable enough to operate in the scenarios mentioned above. In this sense, we propose a sensor fusion algorithm that integrates different sensors (cameras and a robotic total station) in order to increase accuracy and become tolerant to sensor failures. Our system provides a time-persistent localization in the sense that the global coordinates of the inspection points match between different flight operations with a bounded error. This is essential to have the capacity to revisit the same defects periodically (e.g., several months later). More specifically, we present our methodology for UAV localization, our system architecture fusing measurements from heterogeneous sensors, and an implementation in an actual aerial platform.

### 1.1. Related Work

UAV photogrammetry is a common technique that has been used in recent times to inspect and study the state of conservation of bridges and viaducts without the need for an accurate autonomous localization [15,16,17,18]. The photogrammetry process consists of taking overlapping photographs of the object or structure to inspect, and converting them into 3D digital models. This process is carried out in several steps: collecting data, building 3D models, and taking measurements. However, the involved image post-processing is quite expensive computationally, and it has to be done offline. By default, the evolution of the detected defects cannot be traced with this method since it does not establish a time-persistent reference frame. This can be solved, using extra targets with an absolute position; however, this solution is usually cumbersome, subject to errors and cannot be used in real time.

In terms of localization, vision- and LIDAR-based systems are frequently used in aerial robotics applications where the GNSS localization is unreliable. There are popular SLAM (*Self Localization And Mapping*) solutions, such as VSLAM [19], LOAM [20], or ORB-SLAM [21], which aim to solve the localization problem, using onboard sensors. UAV localization in GNSS-denied environments has also been approached by using algorithms based on optical flow and rangefinder sensors [22]. Unlike LIDARs, cameras can offer a more lightweight solution than most mechanical and solid-state LIDARs, which have a weight that is one or even two orders of magnitude greater. This weight issue is particularly relevant for UAV inspection applications, due to the risk reduction required by the UAV regulations. A common problem with these SLAM algorithms is that they only provide a relative localization, using a coordinate frame that typically depends on the starting take-off position. This constraint imposes difficulties in terms of time-persistent localization and structural defect traceability, which are key features to assess how defects evolve over time between inspection operations. Conversely, it is desirable to have a fixed, time-invariant coordinate frame, as this would allow the re-localization of the detected defects, and a proper analysis of the evolution of the global structural health over time, without depending on the initial position and orientation of the robotic platform.

Several works have performed absolute localization without drifting over time [23]. Those works usually use map-matching, a method for self-localization in which sensor data from the local environment are matched with a previously stored map. For instance, the authors in [13] perform pre-flight 3D mapping, and then, they use the *Iterative Close Point* (ICP) algorithm for global localization, also integrating measurements from a visual–inertial odometry method. However, ICP-based algorithms are computationally expensive since they need to process a large amount of data to find a match with the stored map. Thus, they have more difficulties providing real-time localization, which is essential in inspection operations, where UAVs are close to the structure and any small misalignment (arc-minutes) could compromise their safety.

Another approach to obtain a time-persistent coordinate frame is proposed in [24]. They present a localization system based on a total station to inspect bridges with a UAV. A total station is an advanced device commonly used in topography engineering and construction to provide high-resolution position measurements of the targets that the sensor points to. Most frequently used targets can be reflecting stickers for fixed points or reflector prisms for moving objects. A tracking total station (also known as a robotic total station) is a motorized instrument that is able to track autonomously the reflector prism mounted on a moving object. If a reflector prism is mounted on the frame of a UAV, the robotic total station will be able to estimate its position and transmit it to the UAV for navigation and control. However, using a total station as a standalone localization sensor has several drawbacks. First, the reflective target onboard the UAV has to be always in line-of-sight with the total station, which constrains the overall capabilities of the inspection system. Second, the system highly relies on a wireless link between the total station server and the UAV: if that communication link fails, the UAV will lose its position estimation for some time, which is critical for operational safety. Third, the low and variable frequency of the total station measurements (from 0.1 Hz up to 20 Hz) also limits the agility and maneuverability of the UAV.

We partially addressed some of these drawbacks in our previous work [25], where we performed localization for an inspection UAV, combining measurements from a robotic total station and an Intel RealSense T265 camera. This camera comes with an internal processor, where an embedded visual SLAM algorithm [26] can be run. Thanks to the integration of both sensors, we managed to provide a high-frequency localization estimation. Moreover, it was possible to fly beyond the line of sight of the total station, maintaining a good position estimation. Nevertheless, that method relied on an offline camera calibration process that has to be done before flying. Therefore, the system was not capable of correcting online possible misalignments in the calibration, which could have produced relevant localization errors when the UAV is far from the origin of coordinates.

Finally, as a summary, Table 1 shows the main features addressed by each of the approaches discussed in this section, including our new method.

### 1.2. Contributions

In this paper, we present a novel localization system for UAVs oriented to perform inspection operations in civil infrastructures. We improve our previous work [25] by combining measurements from multiple onboard cameras and a robotic total station, as well as devising a new method for online camera recalibration. We have achieved a localization system that is capable of providing a high-frequency and reliable full-state estimation in a time-persistent reference frame, fusing information from heterogeneous sources. Our main contributions are as follows:

- We propose a system architecture (Section 2) where a total station is integrated together with multiple onboard cameras for UAV localization. The addition of multiple cameras enables a wider field of view, which leads to more robust and accurate localization, due to the detection of more visual features.
- We present a state estimation algorithm (Section 3) to align and fuse measurements from heterogeneous sensors, and provide a high-frequency state estimation. The method is based on a stochastic filter that can deal with measurements coming at different rates, and with faulty sensors. This allows us to obtain a reliable state estimation, even when the robotic total station cannot provide accurate measurements, due to issues in the wireless connection or a loss of visibility of the UAV. Our state estimator produces a global, time-persistent localization frame, and it is capable of re-calibrating and re-aligning online the reference frames for the camera-based estimations.
- We have tested our localization system with a real UAV inspecting a viaduct. We present our custom aerial platform and the experimental results (Section 4), as well as our final conclusions (Section 5).

## 2. System Design

**Figure 2:**
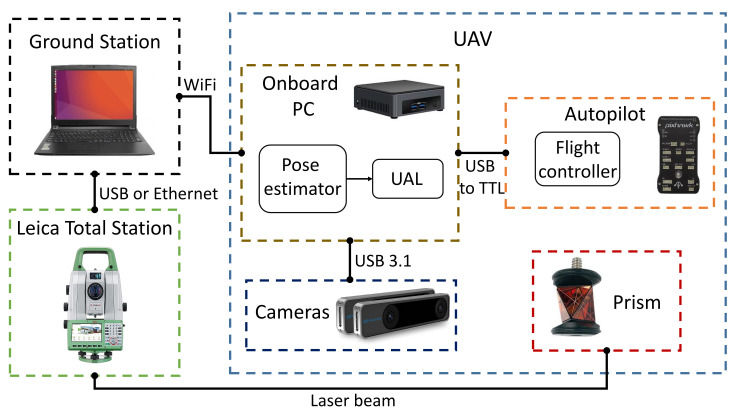
System architecture. The UAV localization is obtained by fusing observations from multiple onboard cameras and a total station on the ground.

Figure 2 illustrates our overall system architecture, depicting the integrated hardware and software components, and their interconnections. We propose a framework that combines observations from several cameras and a robotic total station, with the objective of achieving more reliable and accurate localization.

In the particular implementation of this paper, we have used two RealSense T265 cameras with different configurations onboard the UAV. The main advantages of these devices are the following: they include a lightweight stereo camera with a hemispherical $163\pm 5^{\circ }$ Field of View (FOV), they have low power consumption, and they integrate an embedded visual SLAM solution out of the box [26]. Each camera provides estimations of position, velocity, orientation, and angular velocity, relative to a local reference frame placed on its starting pose. Nonetheless, our fusion algorithm is agnostic to hardware specifics, and it can be adapted to use alternative cameras in the case that a higher performance is required for the target scenario.

Additionally, the information from the cameras is combined with measurements from a Leica MS50 robotic total station, thanks to a GRZ101 mini-prism reflector that is placed on board the UAV. The total station can measure with cm accuracy while flying, and mm accuracy while the UAV is hovering [24]. These measurements are sent to a ground station, which communicates with the UAV onboard PC (Intel NUC) via Wi-Fi. This computer runs the state estimator described in Section 3, which integrates the measurements from all the sensors. The UAL (*UAV Abstraction Layer*) [27] component is a custom layer to interface with the UAV autopilot and sends the corresponding control commands, that uses the mavros interface to communicate with the autopilot.

This proposed system allows us to fulfill our design requirements. First, the accuracy and the time-persistent reference frame can be guaranteed by the robotic total station [24]. However, this sensor is not reliable nor fault-tolerant on its own, due to the variable frequency of its measurements and its faulty wireless communication link. Therefore, the required level of reliability is achieved by also integrating measurements from the onboard cameras, through the state estimation algorithm. The cameras can still provide a state estimation relative to their local reference frames when the UAV is BVLOS of the total station. Then, the state estimator is capable of transforming from these local frames to the static frame of the total station, in order to fuse measurements coming from all sources online. Moreover, this fusion component is tolerant to faulty sensors, as it supervises the quality of each measurement, so that those with big divergences are detected and not integrated into the final estimation. In other words, our state estimator creates a global, time-persistent reference frame linked to the infrastructure; then, the frames of the other sensors are aligned with this global frame through a constant online re-calibration procedure so that all state estimations can be merged together.

## 3. State Estimator

Our state estimation algorithm calculates the full UAV pose estimation (3D position, orientation, linear velocity, and angular velocity), based on the measurements from different sensors.

**Figure 3:**
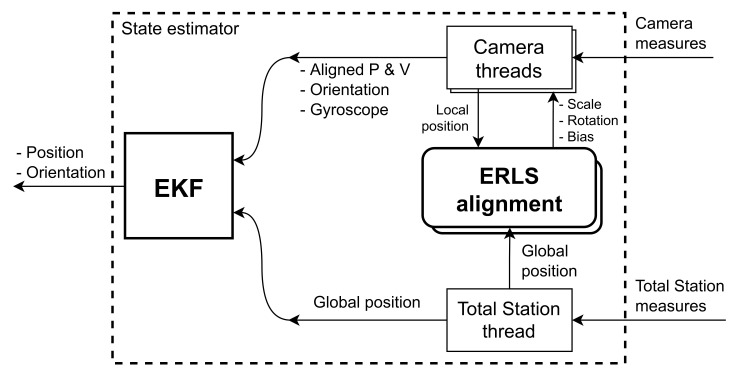
Overall architecture of the state estimator.

Figure 3 shows the overall architecture of our state estimation procedure. The data from the different sensors are processed by an *Extended Recursive Least Squares* (ERLS) alignment module, so that the 3D poses and velocities estimated by the cameras are aligned with the global reference frame provided by the robotic total station. Then, using an *Extended Kalman Filter* (EKF), the aligned position, linear velocities, orientation, and angular velocities estimated by the cameras are fused with the global position provided by the robotic total station, resulting in a final state estimation.

This section describes in detail the different parts of the state estimator. First, it presents the sensors alignment problem and a solution to recursively estimate their best alignment. Second, it describes the used EKF for data fusion and state estimation. Third, it shows how to deal with some known sensor issues. Finally, it details the implementation of the whole system.

### 3.1. Sensors Alignment

As it was proposed in [25], we set a global reference frame {G} linked to the infrastructure, provided by the robotic total station, and a set of local reference frames $\left\{L_{i}\right\}$, which depend on the UAV initial position and orientation, and which are associated with the state estimation algorithm of each camera *i*. We call *alignment* the procedure to express camera-based position and velocity estimations in the global reference frame, by applying the translation and orientation of $\left\{L_{i}\right\}$ with respect to {G}. From now on, to make the notation simpler, we will use *L* instead of $L_{i}$ to denote the local reference frame of any of the cameras since the procedure is the same for all of them.

In our previous work [25], we computed the alignment of the camera measurements, using a least square fitting algorithm, after collecting data and solving the following linear system:

(1) $$p^{G}=T\;p^{L}+p_{0}^{G},$$

where $p^{L}$ and $p^{G}$ are 3D positions expressed in the camera and global reference frames, respectively; *T* is the 3 × 3 transformation matrix; and $p_{0}^{G}$ is the initial UAV 3D position expressed in {G}, provided by the robotic total station. Even though that system is linear, and hence, easier to solve, the method in [25] cannot assure the orthogonality of the resulting reference frame axes, leading to inaccuracies in the final transformation. Here, we solve this problem by defining the following non-linear system:

(2) $$p^{G}=g\left(s,\;q,\;b,\;p^{L}\right)=R\left(q\right)\;S\left(s\right)\;p^{L}+b,$$

where R(q) is the 3 × 3 rotation matrix from {L} to {G}, corresponding to the 4D quaternion q; S(s) is the 3 × 3 scale diagonal matrix, whose diagonal is the 3D scale vector s; and b is a 3D bias vector. The scale vector s would be unitary if the camera had always enough points to perform the visual–inertial state estimation correctly. However, this is not always possible, due to bad light conditions or little visual features. Not only does the bias vector b account for the initial UAV position, but also for the camera drift. We recalculate that drift during the flight, as explained in the following section.

### 3.2. Extended Recursive Least Squares (ERLS) for System Identification

We recursively estimate the alignment vectors q, s and b, in Section 3.1, using a recursive model identification algorithm. During the flight, the UAV collects 3D positions from both local ($p^{L}$) and global ($p^{G}$) sensors, and it calculates the alignment vectors that minimize the mean quadratic error along the trajectory between the measured and the estimated values. The specific algorithm used for this is an extension of the *Recursive Least Squares* (RLS) with the forgetting factor [28]. RLS is a well-known algorithm for system identification in linear systems, i.e., to estimate the parameters of a certain model, given the system inputs and outputs. However, our problem requires a non-linear solution. Thus, we extend the conventional RLS algorithm to deal with non-linear systems.

The original RLS algorithm estimates at each iteration *k* a parameter vector ${\hat{\theta }}_{k}$ such that it minimizes the quadratic error between the measured and the estimated system outputs, along the trajectory from the initial time step t=0 to the current step t=k. Thus, the following summation is minimized:

(3) $$J_{k}\left({\hat{\theta }}_{k}\right)=\sum_{t=0}^{k}\lambda^{k-t}\;{\left(y_{t}-M_{t}{\hat{\theta }}_{k}\right)}^{2},$$

where $y_{t}$ is the measured output at time step *t*, $M_{t}$ is the regressor matrix, and λ∈(0,1] is the forgetting factor. If λ=1, then all errors are considered equally. As we decrease λ, initial errors have less weight in the minimization process.

In our case, the system to identify is non-linear (2), and the parameter vector ${\hat{\theta }}_{k}$ contains the alignment vectors: the scale vector s, the rotation quaternion vector q, and the bias vector b. First, we linearize the non-linear function *g*, using the first-order Taylor series around the estimated variables (variables with and without the hat indicate estimated and actual values, respectively):

(4) $$p_{k}^{G}\approx g\left({\hat{s}}_{k},\;{\hat{q}}_{k},\;{\hat{b}}_{k},\;p_{k}^{L}\right)+J_{b,k}\;\left(b_{k}-{\hat{b}}_{k}\right)+J_{s,k}\;\left(s_{k}-{\hat{s}}_{k}\right)+J_{q,k}\;\left(q_{k}-{\hat{q}}_{k}\right),$$

where $J_{\theta ,k}$ are Jacobian matrices with their components defined by the following:

(5) $$J_{\theta ,k}\left(i,j\right)=\frac{\partial g_{i}}{\partial \theta_{j}}\left({\hat{s}}_{k-1},\;{\hat{q}}_{k-1},\;{\hat{b}}_{k-1},\;p_{k}^{L}\right),$$

with θ∈{s,q,b}. In this case, i∈{0,1,2}, i.e., the three position dimensions, whereas j∈{0,1,2} if θ∈{s,b}, and j∈{0,1,2,3} if θ=q.

Quaternions must be unitary, so we need to impose the unitary condition |q|=1 as an additional restriction. We do this by augmenting the vector function *g* with the quaternion norm, and the global position vector with the scalar 1. We call this new augmented function $g^{\prime }$. Using this, we linearize the standard RLS equations in [28] to apply them to our non-linear system. Thus, the final ERLS equations are the following:

(6) $$\begin{array}{cc} L_{\theta ,k} & =P_{\theta ,k-1}\;J_{\theta ,k}^{\prime }\;{\left(\lambda_{\theta }I+J_{\theta ,k}^{\prime }\;P_{\theta ,k-1}\;J_{\theta ,k}^{\prime ⊤}\right)}^{-1}, \end{array}$$

(7) $$\begin{array}{cc} {\hat{\theta }}_{k} & ={\hat{\theta }}_{k-1}+L_{\theta ,k}\;\left[\left(\begin{array}{c} p_{k}^{G}\\ 1 \end{array}\right)-g^{\prime }\left({\hat{s}}_{k},\;{\hat{q}}_{k},\;{\hat{b}}_{k},\;p_{k}^{L}\right)\right], \end{array}$$

(8) $$\begin{array}{cc} P_{\theta ,k} & =\frac{1}{\lambda_{\theta }}\left(I-L_{\theta ,k}\;J_{\theta ,k}^{\prime }\right)P_{\theta ,k-1}, \end{array}$$

where

(9) $$g^{\prime }\left({\hat{s}}_{k},\;{\hat{q}}_{k},\;{\hat{b}}_{k},\;p_{k}^{L}\right)=\left(\begin{array}{c} g({\hat{s}}_{k},\;{\hat{q}}_{k},\;{\hat{b}}_{k},\;p_{k}^{L})\\ \left|q\right| \end{array}\right),$$

and

(10) $$J_{\theta ,k}^{\prime }\left(i,j\right)=\frac{\partial g_{i}^{\prime }}{\partial \theta_{j}}\left({\hat{s}}_{k-1},\;{\hat{q}}_{k-1},\;{\hat{b}}_{k-1},\;p_{k}^{L}\right).$$

We use these equations to estimate online the best alignment for each moving reference frame. The forgetting factors for each alignment vector, i.e., $\lambda_{s}$, $\lambda_{q}$, and $\lambda_{b}$, are key hyper-parameters that affect the algorithm performance.

### 3.3. Extended Kalman Filter

After the computation of camera alignments, an *Extended Kalman Filter* (EKF) [29] is in charge of fusing the camera measurements (consisting of position, orientation, linear velocity, and angular velocity) with the global position provided by the robotic total station, in order to provide a high-rate accurate estimation of the UAV state in the global reference frame.

The EKF equations to estimate the state vector $x_{k}$ and the covariance matrix $P_{k}$ for each iteration *k* are, for the prediction step, the following:

(11) $$\begin{array}{cc} x_{k}^{-} & =f(x_{k-1}), \end{array}$$

(12) $$\begin{array}{cc} P_{k}^{-} & =F_{k}P_{k-1}F_{k}^{⊤}+Q_{k}, \end{array}$$

and for the update step, the following:

(13) $$\begin{array}{cc} K_{k} & =P_{k}^{-}H_{k}^{⊤}{\left(H_{k}P_{k}^{-}H_{k}^{⊤}+R_{k}\right)}^{-1}, \end{array}$$

(14) $$\begin{array}{cc} x_{k} & =x_{k}^{-}+K_{k}\left[z_{k}-h\left(x_{k}^{-}\right)\right], \end{array}$$

(15) $$\begin{array}{cc} P_{k} & =\left(I-K_{k}H_{k}\right)P_{k}^{-}, \end{array}$$

where *Q* and *R* are the process and measurement noise covariance matrices, respectively, and *F* and *H* are the Jacobian matrices of the prediction and observation functions *f* and *h*, respectively. *K* is the Kalman gain, and z is the measurement vector. Subscript *k* refers to the time step. We define the UAV state vector as follows:

(16) $$x={\left(\begin{array}{cccc} p^{⊤} & v^{⊤} & q^{⊤} & \omega^{⊤} \end{array}\right)}^{⊤},$$

with p as the 3D UAV position, v the 3D linear velocity, q the 4D orientation quaternion, and ω the 3D angular velocity expressed in the UAV body reference frame.

The measurement noise covariance matrices *R* in the EKF are hand-tuned, according to the accuracy of each sensor. In particular, the used values are in the order of $10^{-4}\;I$ for observations from the robotic total station, $10^{-2}\;I$ for position and velocity observations coming from the cameras, and $10^{-4}\;I$ for the orientation and angular velocity measurements from the cameras. Moreover, for the process noise covariance, we found that a value of *Q* of the order of $10^{-5}\;I$ provides a good trade-off between the response time and smoothness in the final state estimation.

The prediction model that relates the current state with its previous value is the following:

(17) $$x_{k}=f\left(x_{k-1}\right)+w_{k-1},$$

where $w_{k-1}$ denotes the process error and *f* is the time evolution function given by the following:

(18) $$f\left(x_{k-1}\right)=\left(\begin{array}{c} p_{k-1}+dt\;v_{k-1}\\ v_{k-1}\\ \Omega_{k-1}\;q_{k-1}\\ \omega_{k-1} \end{array}\right).$$

Here, dt is the sample time, and Ω a 4 × 4 square matrix that represents the quaternion time propagation as a function of $\omega ={\left(\begin{array}{ccc} \omega_{1} & \omega_{2} & \omega_{3} \end{array}\right)}^{⊤}$. This matrix is defined as follows:

(19) $$\Omega =I_{4}+\frac{dt}{2}\;\left(\begin{array}{rrrr} 0 & -\omega_{1} & -\omega_{2} & -\omega_{3}\\ \omega_{1} & 0 & \omega_{3} & -\omega_{2}\\ \omega_{2} & -\omega_{3} & 0 & \omega_{1}\\ \omega_{3} & \omega_{2} & -\omega_{1} & 0 \end{array}\right),$$

and it can be deduced expressing the quaternion differentiation equation as a matrix product:

(20) $$q_{k}=q_{k-1}+\frac{dt}{2}q_{k-1}⊗{\bar{\omega }}_{k-1},$$

where ⊗ denotes quaternion product, and $\bar{\omega }$ is a quaternion whose scalar part is 0 and its vector part ω [30].

The observation model relates the current system state with a measurement from a sensor as follows:

(21) $$z_{k}=h\left(x_{k}\right)+u_{k},$$

with $u_{k}$ the measurement error. The random variables w and u are assumed to be white noises with normal probability distributions:

(22) $$\begin{array}{c} p(w)∼N(0,Q), \end{array}$$

(23) $$\begin{array}{c} p(u)∼N(0,R). \end{array}$$

The update step of our EKF is asynchronous because the sensors provide their measurements at different rates. For this reason, we split the observation function in several parts, which are used depending on the content of each received measurement:

(24) $$\begin{array}{cc} h_{p}\left(x_{k}\right) & =\left(\begin{array}{cc} I_{3} & 0_{3\times 10} \end{array}\right)\;x_{k},\\ h_{v}\left(x_{k}\right) & =\left(\begin{array}{ccc} 0_{3\times 3} & I_{3} & 0_{3\times 7} \end{array}\right)\;x_{k},\\ h_{q}\left(x_{k}\right) & =\left(\begin{array}{ccc} 0_{4\times 6} & I_{4} & 0_{4\times 3} \end{array}\right)\;x_{k},\\ h_{\omega }\left(x_{k}\right) & =\left(\begin{array}{cc} 0_{3\times 10} & I_{3} \end{array}\right)\;x_{k}. \end{array}$$

### 3.4. Fault Tolerance

Our system is prepared to deal with certain identified failures in the sensor data. A first issue is the case when the system has not been receiving measurements from the total station for a significant period of time. This could be caused either by the occlusion of the prism or by a failure in the communication link. When this period with no data from the total station is long, the position provided by the visual–inertial algorithm of the cameras is not corrected for a while, and hence, it might drift. Once the total station is recovered after some time, as it has a high precision, the EKF will correct its position estimation instantly, producing a big leap in the estimation. This effect can negatively affect the UAV controller and produce curtly movements, which could be dangerous when flying close to the structure being inspected. We solve this problem by increasing the covariance *R* associated with measurements from the total station when the time with no observations exceeds a certain threshold, and reducing it back to its default value smoothly when the total station is available again. This is achieved by using first-order dynamics with a time constant τ, selected so that the total station covariance reaches the 99.3% of the final value within 5 s.

Another common failure that the system can address is the case when the visual–inertial state estimation of any of the cameras fails and even diverges. Fusing this erroneous data with the EKF could induce huge errors in the final estimation, endangering the safety of the operation. Therefore, the system is capable of detecting this malfunction and discarding the measurements coming from the erratic sensor. The detection is implemented as follows. First, the received values from the visual–inertial state estimation algorithm should be finite and valid numbers. Otherwise, they are automatically discarded. Second, visual–inertial estimation algorithms usually provide an error covariance that depends on their performance, and that can be used to detect failures. However, the experiments pointed out that these covariance measurements are not always accurate and, before a non-valid value or a big error can be detected, the visual–inertial estimation may have significantly diverged from the actual UAV position and velocity, thus integrating erroneous measurements into the EKF. We solve this by checking the velocity estimation instead. If this estimation is greater than the maximum allowed velocity for the UAV (e.g., in our case, we set a maximum speed of 2 m/s in the autopilot), the corresponding sensor measurements are considered to be erroneous and not integrated into the EKF. We tested both fault-tolerance procedures in real experiments, as shown in Section 4.

### 3.5. Implementation

As it was shown in our architecture in Figure 3, we implement our state estimator with three parallel threads. The first thread executes the prediction step of the EKF at a constant rate of 100 Hz, using Equations (11) and (12) (see Algorithm 1). The second thread corresponds to the total station and it is executed when a measurement from that sensor arrives (Algorithm 2). This thread calculates the alignment parameters and fuses the corresponding position observation into the EKF. Finally, there are additional threads to handle the interface with each of the cameras (Algorithm 3). Each camera thread is executed when new observations from the corresponding camera arrive. All these threads are detailed below.

**Algorithm 1:** EKF prediction thread.

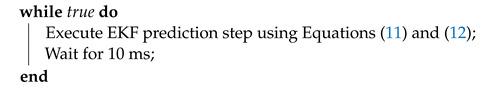



**Algorithm 2:** Total station thread

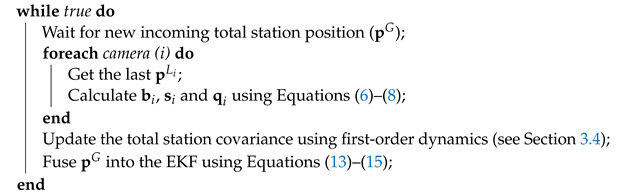



**Algorithm 3:** Camera threads (one for each camera).

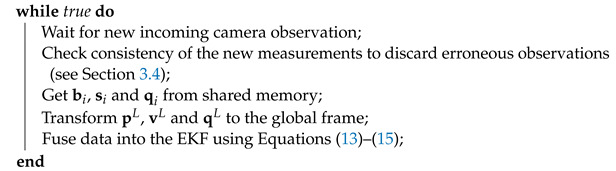



## 4. Experimental Results

In this section, we present the results of a series of inspection experiments to assess the performance of our localization system. First, we conducted tests in a controlled environment to demonstrate all the modules working together in outdoors conditions, and to tune the system. Then, we performed experiments on a real viaduct, where the conditions were more challenging, mainly due to the higher altitude of the operation and the lack of good visual features for the camera visual algorithms. The main goals of our experiments are to demonstrate the following:

- The integration of the whole system, with all components working on our aerial platform.
- Its feasibility for UAV localization in an inspection scenario without GNSS proper coverage.
- The performance of the localization method in terms of accuracy and tolerance to sensor failures.

In the following, we first describe the hardware specifics of our aerial platform and the experimental setup. Then, we present our results in several outdoor experiments, including the performance of the system inspecting a real viaduct. Finally, we discuss about the limitations of the used hardware.

### 4.1. Platform Description

**Figure 4:**
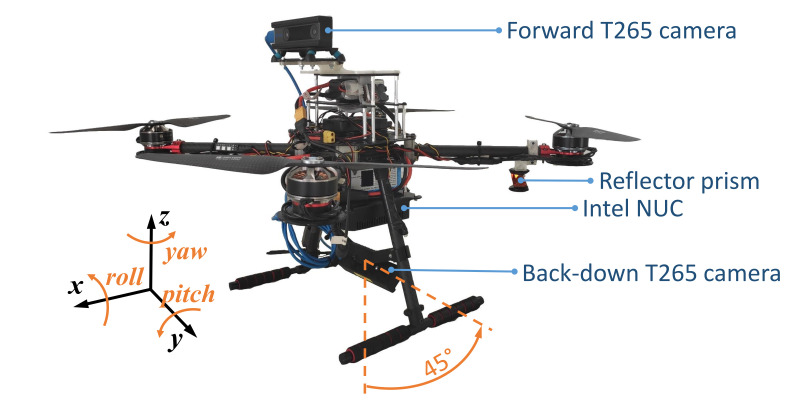
Aerial platform with its different components. One camera is pointing forward and the other one downward, with a 45${}^{\circ }$ angle to the back.

Figure 4 shows the used UAV in our experiments. This platform is a quadrotor based on the Tarot LJI 500-X4 airframe, equipped with four 12-inch propellers, and with a maximum payload of 2 kg. The UAV also has a Pixhawk 1 autopilot [31] that runs the PX4 firmware for flight control [32], and an Intel NUC i7 as the core onboard computer. Following the architecture described in Section 2, a C++ version of our state estimator (Section 3) runs on the onboard computer, fusing measurements from two Intel RealSense T265 cameras and a Leica Nova MS50 robotic total station. We use the API provided by the Intel RealSense SDK 2.0 library [33] to initialize and access the data from the T265 cameras. Our ground station uses a custom client–server software layer to interface with the Leica total station through a serial port and forward the observations to the UAV via Wi-Fi. The communication between the on-board computer and the Pixhawk 1 autopilot is via serial port, and it is completely transparent using UAL. The pose estimate is transferred to the autopilot using the vision input of PX4, writing it in the correspondent mavros ROS topic. All the experiments were performed in autonomous mode, commanding waypoints to the platform using the UAL service *go_to_waypoint*.

Our estimation system could also be used with other closed-source drone solutions, such as the DJI A3. However, due to this, autopilot does not allow to feed the estimator with an external estimation, it would be necessary to develop a higher-level control using this localization and feed the autopilot with velocity or attitude commands through their own SDK.

**Figure 5:**
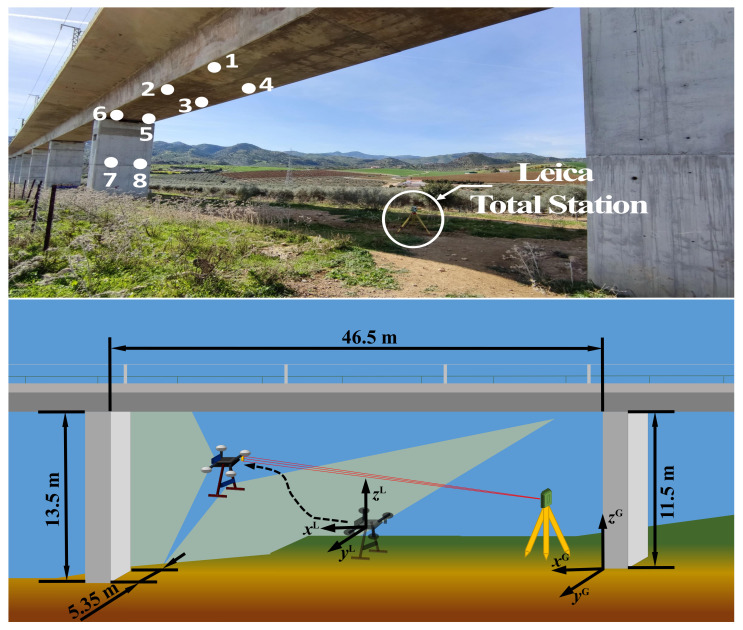
Top: a picture of the viaduct scenario, where the position of the total station and the inspection points are indicated. Bottom: a scheme with measurements of the scenario, the global reference frame, and the camera local reference frame. The red line represents the total station laser beam and the light-green regions the FOV of the cameras.

For these experiments, we choose a camera configuration with one of them pointing forward and the other one looking downward, with an inclination of $45^{\circ }$ to the back (see Figure 4). After some initial tests, this configuration turned out to be the most suitable one in the viaduct scenario (see Figure 5), as it maximized the number of visual features within the field of view of the cameras during the whole inspection flight, thus improving the robustness of the localization system.

### 4.2. Experimental Setup

In all the experiments, we placed the Leica total station at a fixed position on the ground in order to establish the global reference frame. We calibrated that position before each mission by triangulating the total station with several fixed points located in the structure to inspect, having the same global reference frame on different days. We usually placed the total station in such a way that the *X* axis of its reference frame coincides with the longitudinal direction of the inspected structure, the *Y* axis with the transverse direction, and the *Z* axis up, thus facilitating the monitoring of the mission through a better identification of the inspected points.

Regarding the hyper-parameters of the state estimator, we set all forgetting factors ($\lambda_{q}$, $\lambda_{s}$ and $\lambda_{b}$) to 1. Since the T265 cameras may produce pose drifts over time, the bias forgetting factor ($\lambda_{b}$) was initially decreased to weight recent samples more than past samples, and compensate for those drifts. However, we eventually realized in our experiments that a value of 1 yielded better system behavior.

### 4.3. Experiment 1—Mockup Scenario

**Figure 6:**
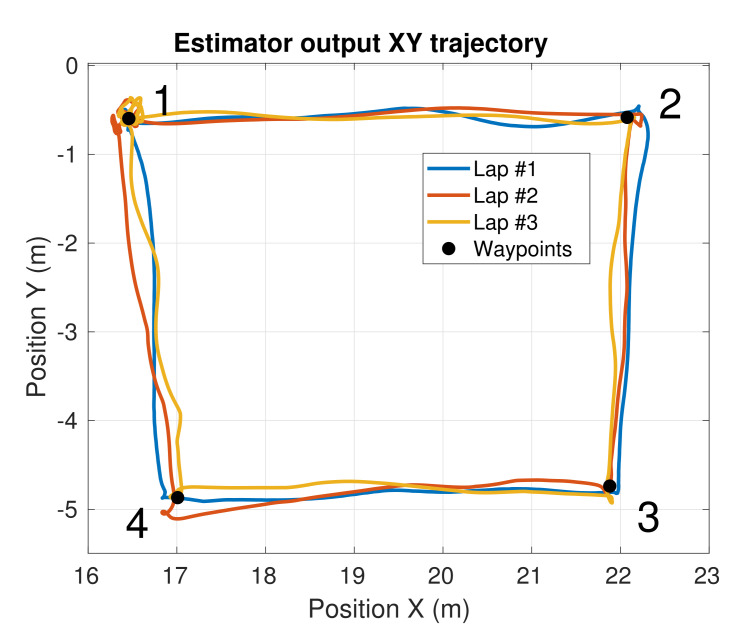
Top view of the estimated UAV trajectory during Experiment 1. The four inspection waypoints are indicated, and each lap is represented with a different color.

**Figure 7:**
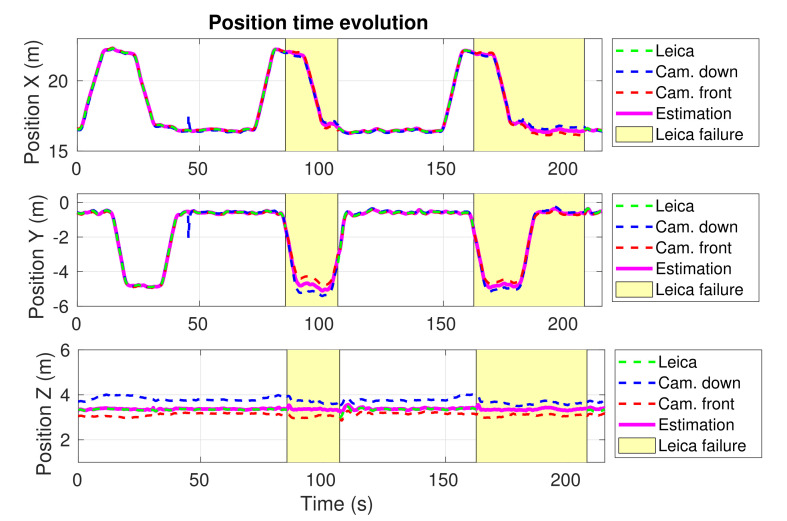
Experiment 1. Time evolution of the estimated X, Y and Z UAV positions provided by our localization system (magenta), the two calibrated camera measurements (blue and red), and the Leica total station (green). The intervals without total station data are shown in yellow.

We performed several tests in a mockup scenario located in the outdoor facilities of our lab. In this experiment, we designed a specific inspection route that was repeated three times by our UAV. This route consists of a squared trajectory given by four inspection waypoints at the corners. Figure 6 shows a top view of the trajectory performed by the UAV while visiting the four corners of the square. During the first lap, all the sensors were working uninterruptedly. In the second lap, the total station server was stopped at t=85 s and recovered at t=107 s, simulating a wireless connection failure. In the last lap, the total station laser beam was occluded at t=162 s and recovered at t=208 s, simulating a temporal loss in the line of sight. Figure 7 shows the temporal evolution of the 3D position estimation of the UAV during the whole experiment, provided by the our system. The estimations provided separately by each of the calibrated cameras and the total station before the fusion are also depicted. Yellow zones represent intervals when the robotic total station was cut off. As can be seen in Figure 6 and Figure 7, the UAV navigated successfully through the four commanded waypoints, even when the robotic total station failed, which indicates both the advantages of fusing heterogeneous sensors and the system fault tolerance. In terms of accuracy, we simulated failures in the total station because this was the most accurate of our sensors. Even though the exact real trajectory followed by the UAV was not available, we used the total station estimation as ground truth for comparison. Thus, after both failure recoveries (t=107 s and t=208 s), we measured the error magnitude between the position estimated by our system and the total station position, which was below 10 cm. Our localization system provided state estimations at 200 Hz. This demonstrates the accuracy and reliability that our localization system was able to achieve, allowing proper navigation, even in the absence of total station observations.

### 4.4. Experiment 2—Real Viaduct

We carried out additional experiments in an actual inspection scenario, in order to assess the performance of our system in more challenging conditions. In particular, the experiments were performed in *Arroyo del Espinazo*, a railway viaduct near Málaga, in the south of Spain (see Figure 5, top). The inspected area of the viaduct is between 11.5 and 13.5 m high, 46.5 m long, and the pillars are 5.35 m wide. Figure 5 (bottom) shows a scheme with the location and orientation of the global and local coordinate systems, and the waypoints of the viaduct to be inspected. We located the Leica total station in such a way that the *X* axis was aligned longitudinally with the viaduct and the *Y* axis transversely. By means of a triangulation procedure using some fixed points and the total station, we created the global fixed reference frame required by the inspection application and our localization system. That frame is time-persistent and was used to visit the same points in different inspection flights.

**Figure 8:**
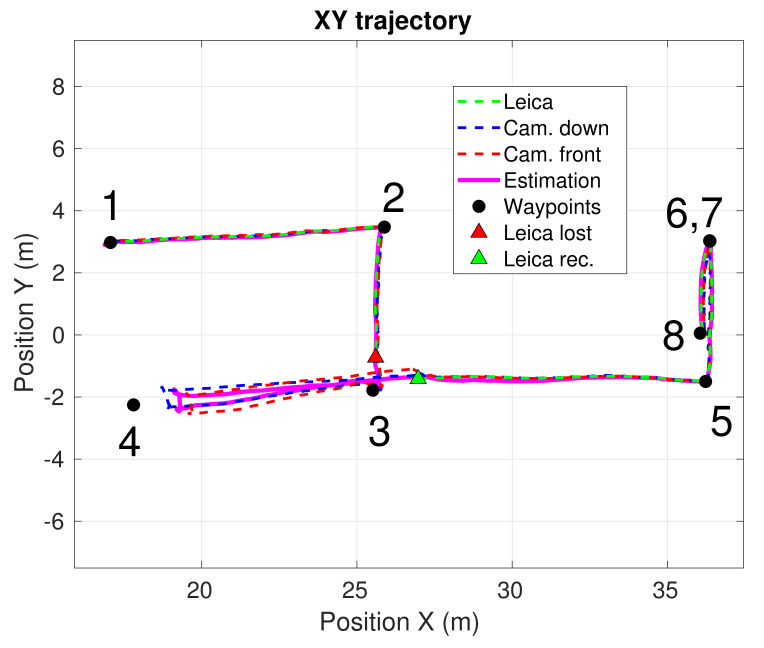
Top view of the estimated UAV trajectory during Experiment 2. The estimation computed by our localization system (magenta), as well as the estimations provided separately by each of the calibrated cameras (blue and red) and the total station (green) before the fusion, are depicted. The additional markers label the inspection waypoints and the points where the total station fails and is recovered, respectively.

**Figure 9:**
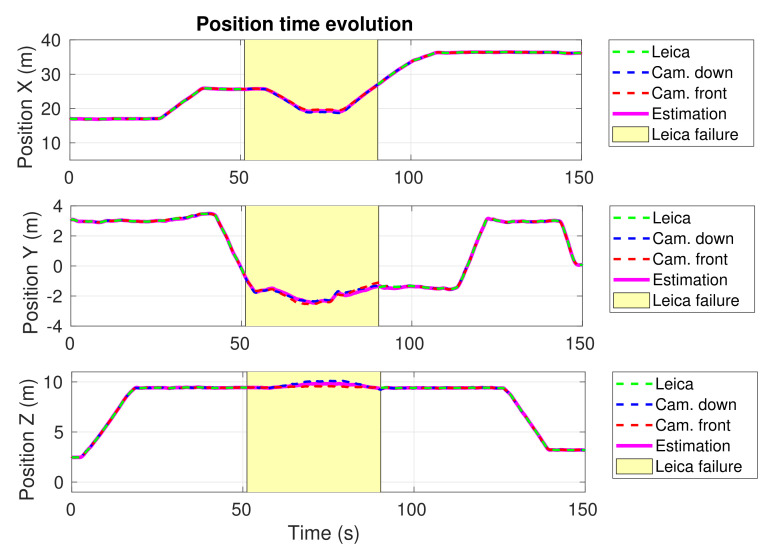
Experiment 2. Time evolution of the estimated X, Y and Z UAV positions provided by our localization system (magenta), the two calibrated camera measurements (blue and red), and the Leica total station (green). The intervals without total station data are shown in yellow.

The inspection mission consisted of visiting autonomously a series of inspection waypoints, located below the deck and on one of the pillars of the viaduct. Figure 5 (top) shows the selected waypoints on a real picture of the infrastructure. As in Experiment 1, we cut off the robotic total station during an interval of the experiment, to test our localization system in the absence of this critical sensor. In Figure 8, a top view of the position estimation is shown, including the estimations computed separately by each of the sensors before sensor fusion. The temporal evolution of the UAV 3D position computed by our system, and without data fusion, are also plotted in Figure 9.

As it can be seen in Figure 8, the estimation provided by our system worsened during the long period without total station measurements (around 40 s), preventing the UAV from reaching the waypoint number 4. This loss of accuracy was caused because the estimation provided by the cameras had a significant error during that period. We experienced that the performance of the visual SLAM running on the RealSense T265 cameras depended on the light exposure, the number of visual features that the cameras were able to detect, and the distribution and distance of these features. Nonetheless, it is important to note that our localization approach is agnostic to the used cameras. Therefore, higher-performance cameras could be integrated for inspection of longer-range scenarios if needed.

**Figure 10:**
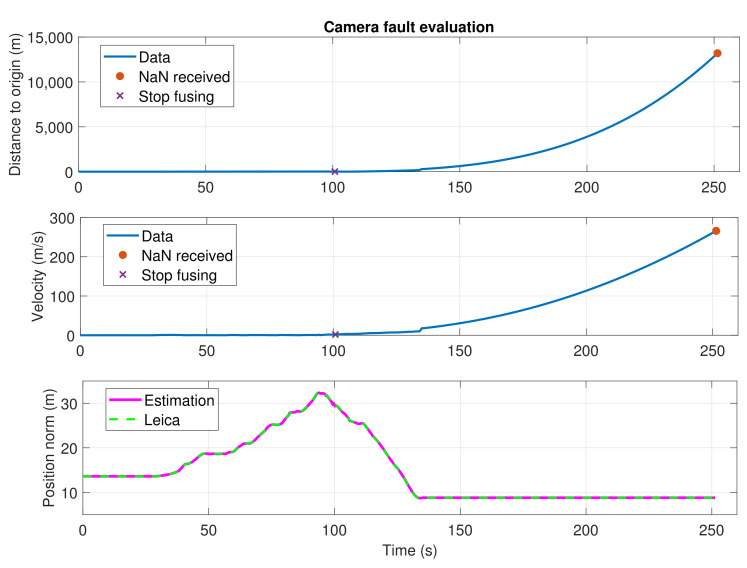
Camera fault evaluation. On top, the measured distance to the origin. In the middle, the velocity norm estimated by the T265 camera algorithm. At the bottom, the position norm, for both our system estimation and the Leica observations.

Finally, Figure 10 shows an experiment where the visual–inertial state estimation algorithm fails. This failure occurred with the downward-back T265 camera in one of the flights performed in the viaduct. At t=103 s, the estimated velocity reaches the maximum limit of 2 m/s, and the algorithm stops fusing observations from that camera, as explained in Section 3.4. After that, the camera measurements continue diverging for more than two minutes, reaching an estimated distance to the origin of 14 km and an estimated velocity of almost 300 m/s. However, the final UAV state estimation does not suffer from those erroneous estimations, thanks to the application of the fault-tolerance procedure. Without this safety feature, the state estimation would have diverged dramatically and the flight would have ended up in an accident.

The results of the experiments in the viaduct demonstrate how the UAV was able to navigate through the inspection points successfully with the localization provided by our method. We proved the capacity of the system to navigate autonomously and safely, even under total station blackouts, thanks to the fusion of heterogeneous sensor sources.

## 5. Conclusions

In this paper, we have presented a localization system for autonomous inspection, using UAVs. We have demonstrated through real experiments that our method can provide reliable and accurate localization in a fixed and time-persistent frame, allowing the traceability of possible defects during subsequent inspection missions. Additionally, we have proved that our system allows navigating safely in the challenging conditions of a real viaduct scenario, being fault-tolerant to the sensor failures.

As future work, we plan to explore the use of alternative sensors to provide more accurate, visual-based estimations in large-scale inspection setups (with up to 100 m height). For cases where a robotic total station cannot be used (e.g., the inspection of a large bridge over a wide river, or inside of a tunnel), we would like to test the possibility of including other accurate localization sensors, such as 2D or 3D LIDARs. Additionally, we would like to study in detail the reliability and the robustness of the classical VSLAM techniques in those kinds of scenarios.

## Figures and Tables

**Table 1:** Related work on localization systems for inspection applications. We indicate if defect traceability is enabled by a time-persistent reference frame, accuracy is enough, reliability is guaranteed by a constant stream at high frequency, the system is tolerant to failures, and it enables a lightweight solution. Where *, **, *** mean minimum, medium and maximun values respectively.

	**Defect Traceability**	**Accuracy**	**Reliability**	**Fault Tolerance**	**Lightweight Solution**
Photogrammetry	*	**	*	*	***
Lidar SLAM	*	**	*	*	*
Visual SLAM	*	*	**	*	***
ICP	**	***	*	*	***
Standalone Leica	***	***	*	*	***
Our approach	***	***	**	***	***
